# ASO Author Reflections: Established Primary Care is Associated with Improved Outcomes in Cancer Surgery

**DOI:** 10.1245/s10434-024-16113-y

**Published:** 2024-08-28

**Authors:** Erryk S. Katayama, Timothy M. Pawlik

**Affiliations:** 1 Department of Surgery, The Ohio State University Wexner Medical Center and James Comprehensive Cancer Center, Columbus, OH USA; 2 The Ohio State University College of Medicine, Columbus, OH USA; 3 Department of Surgery, The Urban Meyer III and Shelley Meyer Chair for Cancer Research, Columbus, OH USA; 4 Oncology, Health Services Management and Policy, The Ohio State University, Wexner Medical Center, Columbus, OH USA

## Past

Primary care (PC) plays a pivotal role in healthcare as the initial point of contact for most patients seeking care, offering consistent and longitudinal management of conditions and coordination of preventative and specialist care. However, availability of PC services is not guaranteed for every patient—access to care is limited by a profound shortage of providers that affects tens of millions of Americans.^1^ Continuity of care and management of comorbidities through established PC has been linked to better perioperative outcomes following emergency general surgery, including reduced morbidity and fewer complications.^2^ Other data have suggested that the association of PC and postoperative outcomes may be important for complex surgical procedures.^3^ The mechanisms by which PC mediates these differences are likely multifaceted, including facilitation of healthier lifestyles and earlier detection of malignancies, as well as optimization of comorbidity burden in the preoperative setting.^4^ We sought to the effects of PC relative to such as hepatopancreatobiliary and colorectal operations performed for a malignant indication.

## Present

The utilization of established PC and its association with postoperative outcomes following major gastrointestinal cancer surgery among Medicare beneficiaries was investigated.^5^ Among 63,177 patients who underwent resection of primary hepatobiliary, pancreatic, or colorectal cancer, almost 20% had no documented PC visit in the year before the surgical procedure. Beneficiaries without established PC were more likely to be racial or ethnic minorities and reside in rural areas or areas with higher social vulnerability. Of note, patients with established PC were 14% more likely to achieve a favorable, overall ‘textbook’ outcome with corresponding odds reduction in complications (− 11%), extended hospital stay (− 19%), and mortality (− 13%) after adjusting for confounding factors. These benefits were most profound among patients who had one to five PC visits per year; in contrast, individuals with more than 10 visits annually, presumably patients who required more active management of various serious comorbidities, did not experience additional benefits and had worse perioperative outcomes. Furthermore, patients without PC were less likely to be discharged to independent care and more likely to need advanced care after discharge, such as skilled nursing or home health. Patients with established PC had lower resource utilization and adverse events, which corresponded to a 4–5% reduction in expenditures.

## Future

Patients without established PC are vulnerable populations that are at risk for worse outcomes and increased expenditures. An important component of the healthcare delivery system, PC offers benefits that include improving perioperative outcomes among patients who undergo complex surgical oncology procedures. Addressing the shortage in primary providers is needed to improve access to PC, especially among underprivileged rural residents, as well as traditionally marginalized racial/ethnic minority populations. Improving surgical outcomes requires a focus on the broader continuum of care that begins well before the operating room with PC providers.
